# Evaluation of *BRCA1 *and *BRCA2 *mutations and risk-prediction models in a typical Asian country (Malaysia) with a relatively low incidence of breast cancer

**DOI:** 10.1186/bcr2118

**Published:** 2008-07-16

**Authors:** E Thirthagiri, SY Lee, P Kang, DS Lee, GT Toh, S Selamat, S-Y Yoon, NA Mohd Taib, MK Thong, CH Yip, SH Teo

**Affiliations:** 1 Cancer Research Initiatives Foundation, Subang Jaya Medical Centre, Kuala Lumpur, Malaysia; 2 University Malaya Medical Centre, Kuala Lumpur, Malaysia

## Abstract

**Introduction:**

The cost of genetic testing and the limited knowledge about the *BRCA1 *and *BRCA2 *genes in different ethnic groups has limited its availability in medium- and low-resource countries, including Malaysia. In addition, the applicability of many risk-assessment tools, such as the Manchester Scoring System and BOADICEA (Breast and Ovarian Analysis of Disease Incidence and Carrier Estimation Algorithm) which were developed based on mutation rates observed primarily in Caucasian populations using data from multiplex families, and in populations where the rate of breast cancer is higher, has not been widely tested in Asia or in Asians living elsewhere. Here, we report the results of genetic testing for mutations in the *BRCA1 *or *BRCA2 *genes in a series of families with breast cancer in the multi-ethnic population (Malay, Chinese and Indian) of Malaysia.

**Method:**

A total of 187 breast cancer patients with either early-onset breast cancer (at age ≤ 40 years) or a personal and/or family history of breast or ovarian cancer were comprehensively tested by full sequencing of both *BRCA1 *and *BRCA2*. Two algorithms to predict the presence of mutations, the Manchester Scoring System and BOADICEA, were evaluated.

**Results:**

Twenty-seven deleterious mutations were detected (14 in *BRCA1 *and 13 in *BRCA2*), only one of which was found in two unrelated individuals (*BRCA2 *490 delCT). In addition, 47 variants of uncertain clinical significance were identified (16 in *BRCA1 *and 31 in *BRCA2*). Notably, many mutations are novel (13 of the 30 *BRCA1 *mutations and 24 of the 44 *BRCA2*). We report that while there were an equal proportion of *BRCA1 *and *BRCA2 *mutations in the Chinese population in our study, there were significantly more *BRCA2 *mutations among the Malays. In addition, we show that the predictive power of the BOADICEA risk-prediction model and the Manchester Scoring System was significantly better for *BRCA1 *than *BRCA2*, but that the overall sensitivity, specificity and positive-predictive value was lower in this population than has been previously reported in Caucasian populations.

**Conclusion:**

Our study underscores the need for larger collaborative studies among non-Caucasian populations to validate the role of genetic testing and the use of risk-prediction models in ensuring that the other populations in the world may also benefit from the genomics and genetics era.

## Introduction

The discovery of the *BRCA1 *and *BRCA2 *genes has transformed the management of women who are at high risk of developing breast and ovarian cancer. Diagnostically, it has facilitated the accurate identification of women at risk of cancer; through screening and preventive measures, it has reduced the incidence of cancer in such women [1], and perhaps most importantly, exploitation of the knowledge that these genes function in DNA repair has opened up avenues for the development of new treatments for women with hereditary tumours [2].

Although predictive genetic testing is increasingly becoming a part of clinical practice in many developed countries, the cost of testing and the limited knowledge about the prevalence of these genes in different ethnic groups has limited its availability in medium- and low-resource countries [3,4]. There is relatively little information about the *BRCA1 *and *BRCA2 *mutations in Asia and among Asians living elsewhere and, to date, no significant founder-effect mutation has been reported [5,6]. Notably, although 60% of the world's population reside in the Asian continent, and the Malays, Chinese and Indians are three major Asian ethnic groups, relatively little is known about the genetic predisposition to hereditary diseases and the applicability of genetic testing in these diverse ethnic groups [7-16].

Malaysia is a multi-ethnic country with a population of approximately 23 million, comprising approximately 65% Malays and other indigenous groups, approximately 26% Chinese and approximately 8% Indian (Malaysia Housing and Population Census 2000, Department of Statistics Malaysia). The Chinese and Indians are largely first- or second-generation migrants from southern China and southern India, while the Malays and other minority indigenous groups are native to South-East Asian countries including Malaysia and Indonesia. In Malaysia, which may be described as a typical Asian country, breast cancer is characterised by: a lower age-standardised rate (30 per 100,000 [17]); a proportionately higher incidence of early-onset breast cancer (47% of new cases occurred in women <50 years [17]); and an incomplete family history or structure because of a lack of information about second degree and further relatives, premature mortality and significant dispersal of families. Taken together, studies in this typical Asian multi-ethnic population provide an excellent opportunity to understand genetic predisposition among the different ethnic groups and among the different risk categories.

Furthermore, in order to ensure that medicine based in genetics can also be equitably implemented in low- and medium-resource countries, it is critically important that there are cost-effective mutation screening programmes. Currently, there is little information about the effectiveness of these risk-assessment tools in other ethnic populations, particularly in countries with low age-standardised rate of breast cancer, which also reflects the setting in many low- and medium-resource countries.

Using a unique, ethnically diverse cohort of high-risk families, we sought to: examine the significance of founder/recurrent and novel rare *BRCA1 *and *BRCA2 *mutations to familial breast and ovarian cancer in the Malays, Chinese and Indian populations of Malaysia; and compare the accuracy of the Manchester Scoring System and the BOADICEA (Breast and Ovarian Analysis of Disease Incidence and Carrier Estimation Algorithm) risk-prediction models to predict pathogenic mutations and particularly to discriminate at the 10% likelihood level. Taken together, we wished to devise the most relevant definition of individuals or families who would benefit from mutation testing in this typical Asian country with a lower incidence of breast cancer.

## Materials and methods

### Breast cancer cohort

The recruitment of Malaysian families with a high risk of breast or ovarian cancer started in 2003 at the University Malaya Medical Centre in Kuala Lumpur. Patients with breast cancer were first approached by clinicians responsible for their care to see if they would participate in a research study to determine the genetic factors which increase the risk of breast cancer in Malaysia. Thereafter, individuals interested in the project were approached by a member of the research team who explained to them the nature and objectives of the research project. A total of 678 index cases were referred to our study between January 2003 and December 2007. Index cases signed a consent form and a blood sample was taken. A family history was recorded and the pedigree analysed. Where possible, pathology reports were requested to confirm all diagnoses of breast and ovarian cancers in the index case.

The study was approved by the ethics committee of University Malaya Medical Centre.

### Inclusion criteria for analysis of *BRCA1 *and *BRCA2*

All breast cancer patients in the cohort had: early-onset breast cancer (≤ 40 years) and 1 or more additional cases of breast cancer in first- or second-degree relatives; breast cancer (≥ 40 years) and two or more additional cases of breast cancer in first- or second-degree relatives; bilateral breast cancer; or a personal or family history of ovarian cancer. They were analysed for mutations in the *BRCA1 *and *BRCA2 *genes. In addition, approximately 50% of patients with only early-onset breast cancer (≥ 40 years) with no significant family history or breast cancer (≤ 40 years) and one additional case of breast cancer in a first- or second-degree relative were also analysed. Individuals were categorised based on self-reported race or ethnicity. Of the 678 index cases, 187 were analysed for mutations in the *BRCA1 *and *BRCA2 *genes (Table 1).

**Table 1:** Family characteristics

	**Total (n = 187)**	**Malay (n = 44)**	**Chinese (n = 118)**	**Indian (n = 22)**	**Others (n = 3)**
	
	**No. (%)**	**No. (%)**	**No. (%)**	**No. (%)**	**No. (%)**
**Age of onset of breast cancer in index case (years)**					

20 to 29	14 (7)	3 (7)	10 (8)	1 (5)	0 (0)
30 to 39	64 (34)	21 (48)	36 (31)	6 (27)	1 (33)
40 to 49	49 (26)	8 (18)	35 (30)	5 (23)	1 (33)
≥ 50	60 (32)	12 (27)	37 (31)	10 (45)	1 (33)

**Personal cancer history of breast, or breast and ovarian cancer**					

Breast only	182 (97)	43 (98)	117 (99)	19 (86)	3 (100)
Both breast and/or ovarian	5 (3)	1 (2)	1 (1)	3 (14)	0 (0)
Bilateral breast cancer	25 (13)	6 (14)	15 (13)	4 (18)	0 (0)

**Family history of breast and ovarian cancer in pedigree**					

1 case (no family history)	^a^73 (39)	24 (54)	40 (34)	8 (36)	1 (33)
2 cases	76 (41)	13 (30)	54 (46)	9 (41)	1 (33)
3 cases	24 (13)	4 (9)	17 (14)	2 (9)	0 (0)
≥ 4 cases	14 (7)	3 (7)	7 (6)	3 (14)	1 (33)

A total of 187 breast cancer patients were analysed for mutations in *BRCA1 *and *BRCA2 *by DNA sequencing and multiple ligation dependent probe amplification (MLPA) analysis. Table 1 shows the distribution of patients according to their self-declared ethnicity, personal history of breast and ovarian cancer, and family history of breast and ovarian cancer in first- and second-degree relatives. ^a^Notably, the 73 individuals with no family history of breast and ovarian cancer were included in the study because they had either early age of onset of breast cancer (≤ 40 years of age: 45 individuals; 41 to 55 years of age: nine individuals), bilateral breast cancer (16 individuals), both breast and ovarian cancer (one individual) or family history of prostate cancer in the third degree (three individuals).

### Mutation detection

Blood from index cases was separated into two 10 ml EDTA-tubes and DNA was extracted using standard methods. Samples were analysed either at Genetic Technologies Laboratory (Australia) or at Cancer Research Initiatives Foundation (Malaysia) using direct DNA sequencing and multiple ligation dependent probe amplification (MLPA), as previously described [18]. Confirmation tests were performed on a second blood sample. Naming and interpretation of sequence analysis were performed as previously described and all patients were classified as having a deleterious mutation if the *BRCA1 *or *BRCA2 *protein terminated prematurely at least 10 or 110 amino acids, respectively, from the C terminus. Genetic variants of undetermined clinical significance (unclassified variants) included missense mutations and mutations that occurred in analysed intronic regions whose clinical significance had not yet been determined. If more than one variant was observed in a single analysis, the overall interpretation was that of the most clinically significant mutation observed.

### BOADICEA

The BOADICEA model allows for a polygenic modifier locus effect in which several low-penetrance genes have joint effects [19]. Family history information (including incidence of breast, ovarian and other cancers, age at diagnosis and relationship to the proband) was collected and recorded by a genetic counsellor or researcher. Breast cancer patients were included in the BOADICEA analysis if they had more than one primary cancer or if they had a family history of breast, ovarian, pancreatic and/or prostate cancer in first-, second- or third-degree relatives. Of the 187 individuals who were analysed for mutations in *BRCA1 *and *BRCA2*, we excluded 42 individuals (including three individuals with deleterious *BRCA2 *mutations), all of whom had a single case of breast cancer and no family history of breast, ovarian, pancreatic or prostate cancer in first-, second- or third-degree relatives. The predicted likelihood of carrying either a *BRCA1 *or *BRCA2 *mutation was generated for each individual using BOADICEA risk estimation on the internet (BOADICEA WEB APPLICATION v1.0).

### Manchester Scoring System

We used the Manchester Scoring System to estimate the probability of identifying mutations in *BRCA1 *and *BRCA2 *genes [20,21]. In brief, the scoring system was developed using a combination of results from screening and family history of those with and without mutations, where a combined score of 15 or more was proposed to correlate with a 10% mutation probability. The exclusion criteria for BOADICEA analysis was also applied to the Manchester Scoring System.

## Results

### Mutation testing outcome

A total of 1226 patients with breast cancer were treated at the University Malaya Medical Centre between January 2003 and December 2007, of which 678 individuals with breast cancer from 674 families were recruited to the present study. Despite rigorous efforts to identify women with a family history of breast cancer, only 159 women were found to exhibit a family history in a first- or second-degree relative (129 with one additional affected member, 19 with two additional affected members and 11 with three or more additional affected members).

We conducted full sequence analysis of the *BRCA1 *and *BRCA2 *genes in 187 women (Table 1). Of these: 73 had no significant family history of breast or ovarian cancer in first- or second-degree relatives (45 of whom developed breast cancer ≤ 40 years old, nine of whom developed breast cancer between 41 and 55 years old; one had both breast and ovarian cancer, 16 had bilateral breast cancer and two had a family history of prostate cancer); and 114 had two or more cases of breast or ovarian cancer in first- or second-degree relatives.

Mutation detection led to the discovery of 27 deleterious mutations in 28 breast cancer patients (14 in *BRCA1 *and 13 in *BRCA2*; Table 2). Notably, 14 of these mutations were novel (five in *BRCA1 *and eight in *BRCA2*) and one mutation in *BRCA2 *was found in two unrelated families. In addition, we identified 47 sequence variants of unknown clinical significance (16 in *BRCA1 *and 31 in *BRCA2*), of which eight were found to occur in more than one unrelated family (Table 3). Of these, 26 sequence variants were novel (nine in *BRCA1 *and 17 in *BRCA2*). Eleven sequence variants (two in *BRCA1 *and nine in *BRCA2*) may be potentially damaging based on sequence conservation and Grantham score, and five sequence variants are unlikely to be clinically relevant.

**Table 2:** Deleterious mutations including frame-shift, nonsense and splice site deleterious mutations identified in Malaysian breast cancer patients

**No.**		**Exon**	**Nucleotide change**	**AA change**	**Reported**	**Family (No.)**	**Ethnicity**
1	*BRCA1*	2	180 delA	STOP 22	**Novel**	1	Indian
2		2	185 delAG	STOP 39	Ashkenazi Jews, others	1	Indian
3		2	185 insA	STOP 40	Caucasian, European	1	Chinese
4		8	589 delCT	STOP 157	Caucasian, Caribbean	1	Chinese
5		11	1100 delAT	STOP 328	Caucasian, Chinese [10,16]	1	Chinese
6		11	1173 G>T	E352X	Caucasian	1	Indian
7		11	1323 G>T	E402X	**Novel**	1	Malay
8		11	3889 delAG	STOP1265	Various	1	Chinese
9		13	4377 C>T	Q1420X	Caucasian, African-American	1	Chinese
10		20	5370 C>T	R1751X	Various	1	Indian
11			IVS 3+1 G>T		**Novel**	1	Chinese
12			IVS 3+2 delT		**Novel**	1	Chinese
13			IVS 4-1G>C		**Novel**	1	Indian
14			IVS 5-12 A>G		Various	1	Chinese

1	*BRCA2*	3	490 delCT	STOP 99	**Novel**	2	Malay
2		10	1184 insA	STOP 326	Caucasian	1	Chinese
3		10	2001 del4	STOP 612	Caucasian	1	Chinese
4		11	2699 del6	STOP 824	**Novel**	1	Malay
5		11	2864 delCT	STOP 879	**Novel**	1	Chinese
6		11	4265 delCT	STOP 1350	Filipino	1	Malay
7		11	6195 insA	STOP 2002	**Novel**	1	Malay
8		11	6553 delGT	STOP 2109	**Novel**	1	Chinese
9		11	6901 delA	STOP 2228	**Novel**	1	Chinese
10		11	6943 G>T	E2239X	**Novel**	1	Chinese
11		22	9097 C>T	Q2957X	**Novel**	1	Indian
12		23	9326 insA	STOP 3042	Various	1	Chinese
13			IVS 17+1 G>A		Caucasian	1	Chinese

**Table 3:** Missense and intervening sequence variants identified

	**No.**	**Exon**	**Nucleotide change**	**AA change**	**Reported**	**Family (No.)**	**Mutation classification**	**Ethnicity**
*BRCA1*	1	7	491 C>A	I124I	**Novel**	1	Benign?	Malay
	2	9	690 G>A	V191I	Various	2	Benign?	Chinese
	3	11	873 C>T	R252C	**Novel**	1	Deleterious?	Malay
	4	11	914 T>C	S265S	**Novel**	1	Benign?	Chinese
	5	11	1155 C>T	P346S	Asian	2	Benign?	Chinese
	6	11	2405 A>T	R762S	Chinese [8]	1	Benign?	Malay
	7	11	2685 T>C	Y856H	Chinese	6	Benign?	Chinese
	8	11	2845 A>T	N909I	Chinese	1	Benign?	Chinese
	9	11	2858 T>A	N913K	**Novel**	1	Benign?	Chinese
	10	11	3050 A>G	P977P	**Novel**	1	Benign?	Chinese
	11	11	3781 A>C	E1221A	**Novel**	1	Benign?	Chinese
	12	11	3922 A>G	N1268S	**Novel**	1	Benign?	Malay
	13	16	5011 G>A	S1631N	**Novel**	1	Benign?	Chinese
	14	24	5623 G>A	R1835Q	**Novel**	1	Benign?	Malay
	15		IVS 1-10 T>C		Various	1	Not clinically relevant [43]	Malay
	16		IVS 12-10 G>A		Various	1	Deleterious?	Indian

*BRCA2*	1	3	443 A>G	N72S	**Novel**	1	Benign?	Chinese
	2	5	668 A>G	Q147R	Asian, various	2	Benign?	1 Malay, 1 Chinese
	3	10	1171 T>A	C315S	Asian	2	Benign?	Chinese
	4	10	1503 A>G	E425E	**Novel**	1	Benign?	Chinese
	5	10	1590 A>G	K454K	**Novel**	3	Benign?	Chinese
	6	10	1828 G>A	E534K	**Novel**	1	Deleterious?	Malay
	7	10	1872 G>A	Q548Q	Various	1	Benign?	Chinese
	8	10	1875 G>A	K549K	**Novel**	1	Benign?	Chinese
	9	10	2053 C>G	Q609E	**Novel**	1	Benign?	Malay
	10	11	2906 A>G	Q893R	**Novel**	1	Benign?	Malay
	11	11	3648 T>C	S1140S	Chinese [8]	1	Benign?	Chinese
	12	11	3673 A>G	M1149V	Asian, various	3	Benign?	2 Malay, 1 Chinese
	13	11	3903 A>G	T1225T	**Novel**	1	Benign?	Malay
	14	11	4010 C>G	S1261C	**Novel**	1	Deleterious?	Malay
	15	11	4806 A>G	T1526T	**Novel**	1	Benign?	Other
	16	11	5395 A>C	T1723P	**Novel**	1	Benign?	Malay
	17	11	5540 G>A	G1771D	Various	1	Not clinically relevant [BIC]	Malay
	18	11	5863 G>A	E1879K	Caucasian	1	Benign?	Indian
	19	11	6013 A>G	I1929V	Asian, various	1	Not clinically relevant [BIC]	Chinese
	20	11	6550 C>T	R2108C	Various	3	Benign?	2 Malay, 1 Chinese
	21	12	7157 C>A	T2310N	**Novel**	1	Deleterious?	Indian
	22	17	8169 A>C	L2647L	**Novel**	1	Benign?	Malay
	23	18	8415 G>T	K2729N	Asian, various	1	Not clinically relevant [44]	Chinese
	24	19	8584 G>A	A2786T	**Novel**	1	Deleterious?	Chinese
	25	21	8930 G>A	G2901D	Asian	1	Deleterious?	Chinese
	26	23	9332 A>G	Y3035C	Caucasian	1	Deleterious?	Chinese
	27	23	9334 C>G	Q3036E	**Novel**	1	Benign?	Chinese
	28	27	10135 A>T	S3303C	**Novel**	1	Deleterious?	Other
	29	27	10462 A>G	I3412V	Various	1	Not clinically relevant [45]	Chinese
	30		IVS 2-7T>A		Caucasian	1	Deleterious?	Malay
	31		IVS 7-10 insT		**Novel**	1	Deleterious?	Other

Mutation classification was made based on published studies and analysis using PolyPhen. BIC, Breast Cancer Information Core.

Table 4 shows that the majority of mutations in the Indian subgroup were *BRCA1 *mutations, whereas the majority of the mutations in the Malay subgroup were *BRCA2 *mutations, and there were an equal number of mutations in both genes among the Chinese subgroup. One possible explanation for the higher incidence of *BRCA1 *mutations in the Indian subgroup is that there was a high proportion of women with both breast and ovarian cancer in this subgroup compared with the Chinese or Malay subgroups (14% of total, compared with 1 to 2% among the Chinese and Malay; Table 1) and it is clear from other studies that *BRCA1 *mutations confer a higher penetrance to ovarian cancer [22]. By contrast, the reason for the higher prevalence of *BRCA2 *mutations among the Malays is unclear.

**Table 4:** Incidence of *BRCA1 *and *BRCA2 *deleterious mutations, by race/ethnicity

**Ethnicity**	**Total families**	**Families with deleterious mutation**		**Families with unclassified variant**	
		
		***BRCA1***	***BRCA2***	***BRCA1***	***BRCA2***
**Malay**	44	1	5	6	14
**Chinese**	118	8	8	15	18
**Indian**	22	5	1	1	2
**Others**	3	0	0	0	3

**Total**	**187**	**14**	**14**	**22**	**37**

### Clinical presentation of breast cancer

The mean age of diagnosis of all women analysed was 43.8 years (range 22 to 78 years), and the mean age of diagnosis was 40.3 years for *BRCA1*-positive (range 28 to 57 years), 43.6 years for *BRCA2*-positive (range 34 to 59 years) and 44.2 years for *BRCA*-negative women (range 22 to 78 years) (Table 5 and 6). This is comparable to data from a large study of 10,000 individuals in the USA [6], where individuals with *BRCA1 *mutations had a significantly younger age at diagnosis (40 years) than those with *BRCA2 *(41 years).

**Table 5:** Family characteristics and pathological characteristics of breast cancers of individuals with deleterious *BRCA1 *and *BRCA2 *mutations

	**Nucleotide change**	**AA change**	**Age of diagnosis of breast cancer**	**No. of breast/ovarian cancers in family**	**Mean age diagnosis (Breast cancer)**	**No. of cases (Breast cancer ≤ 50)**	**ER**	**PR**	**HER2**	**Grade**	
*BRCA1*	180 delA	STOP 22	55	2	43	1	-	-	-	2	IDC
	185 delAG	STOP 39	33	1	33	1	-	-	-	3	IDC
	185 insA	STOP 40	57	7	44	4	NA	NA	NA	NA	IDC
	589 delCT	STOP 157	45	3	41	3	-	-	-	NA	IDC
	1100 delAT	STOP 328	28	2	28	1	-	-	-	3	IDC
	1173 G>T	E352X	45	3	45	1	-	-	-	2	IDC
	1323 G>T	E402X	34	3	31	2	NA	NA	NA	NA	Sarcoma
	3889 delAG	STOP1265	47	3	47	2	-	NA	+	NA	DCIS/IDC
	4377 C>T	Q1420X	39	2	45	2	-	-	+/-	3	IDC
	5370 C>T	R1751X	31	5	38	3	NA	NA	NA	NA	NA
	IVS 3+1 G>T		46	2	47	2	-	NA	+	NA	IDC
	IVS 3+2 delT		32	2	56	1	-	NA	-	2	IDC
	IVS 4-1G>C		39	2	48	1	NA	NA	NA	NA	IDC
	IVS5-12 A>G		33	1	33	1	-	-	-	3	IDC

*BRCA2*	490 delCT	STOP 99	39	2	56	1	-	-	-	2	IDC
	490 delCT	STOP 99	51	4	45	3	+	+	-	NA	ILC
	1184 insA	STOP 326	34	1	34	1	+	+	-	2	IDC
	2001 del4	STOP 612	37	1	37	1	+	-	+	3	IDC
	2699 del6	STOP 824	41	2	57	1	+	NA	NA	2	IDC
	2864 delCT	STOP 879	40	1	40	1	-	NA	NA	3	IDC
	4265 delCT	STOP 1350	55	4	44	3	+	+	-	2	IDC
	6195 insA	STOP 2002	42	3	46	2	NA	NA	NA	NA	NA
	6553 delGT	STOP 2109	59	3	49	1	+	NA	-	2	IDC
	6901 delA	STOP 2228	35	1	35	1	NA	NA	NA	NA	IDC/DCIS
	6943 G>T	E2239X	47	2	44	2	NA	NA	NA	NA	NA
	9097 C>T	Q2957X	38	7	44	2	NA	NA	NA	NA	NA
	9326 insA	STOP 3042	56	4	49	2	-	-	NA	NA	NA
	IVS 17+1 G>A		36	2	46	1	-	-	-	3	IDC

ER, oestrogen receptor; PR, progesterone receptor; AA, amino acid; DCIS, ductal carcinoma *in situ*; IDC, invasive ductal carcinoma; ILC, invasive lobular carcinoma.

**Table 6:** Summary of family characteristics and pathological characteristics of breast cancers of individuals with deleterious *BRCA1 *and *BRCA2 *mutations

	Overall	*BRCA1*	*BRCA2*	Non-*BRCA*
Age of onset	22 to 78	28 to 57	34 to 59	22 to 78
Average age of onset (years)	41.9	40.3	43.6	44.2
Total no. of breast and ovarian cancers in family	1 to 7	1 to 7	1 to 7	1 to 6
Average no. of breast and ovarian cancers in family	1.9	2.7	2.6	1.8
No. of cases of breast cancer <50	0 to 4	1 to 3	1 to 3	0 to 4
Average no. of cases of breast cancer <50	1.1	1.6	1.5	1.0
% with ER-negative tumours	48%	100%	40%	45%

Table 5 shows the pathological characteristics of breast cancer arising in *BRCA1 *and *BRCA2 *deleterious mutation carriers. Consistent with other studies [23], the majority of *BRCA1 *tumours are high grade and negative for the oestrogen, progesterone and *HER2 *receptors. By contrast, the *BRCA2 *tumours arise with various status for the oestrogen, progesterone and *HER2 *receptor.

### Prediction of *BRCA1*/*BRCA2 *carrier status based on risk factors

Table 7 shows the likelihood of having a *BRCA1 *or *BRCA2 *deleterious mutation, based on the personal and family history of the individual. We found that the best predictive factors for the presence of a *BRCA1 *or *BRCA2 *mutation are families with at least two cases of breast cancer, at least one of which occuring before the age of 50 (p = 0.0335), and families with a history of both breast and ovarian cancer (p < 0.0001). Notably, of the 15 breast cancer patients with personal or family history of ovarian cancer, six had mutations in *BRCA1 *and two in *BRCA2*. In addition, of the 14 *BRCA1 *carriers and 14 *BRCA2 *carriers identified in this study, five had bilateral breast cancer (one synchronous and four metachronous). The mean time interval between surgery for the first primary cancer and the second occurrence in the contralateral breast was 9.8 years (range four to 24 years). However, the association between bilaterality and *BRCA *mutation status was not statistically significant (p = 0.45). Taken together, the data suggests that the presence of two or more breast cancers with at least one case under the age of 50, and ovarian cancer at any age are significant predictors for the presence of a *BRCA1 *or *BRCA2 *mutation.

**Table 7:** Likelihood of having a *BRCA1 *or *BRCA2 *deleterious mutations

**Personal history**	**Family history**	**Total**	***BRCA1 *positive**	***BRCA2 *positive**	***BRCA *positive**	***BRCA *positive**
			
			Number	Number	Number	%
Breast cancer <50 years	Nil	61	2	3	5	8.2
Breast cancer <50 years	1 breast cancer <50 years	29	1	3	4	13.8
Breast cancer ≥ 50 years	1 breast cancer <50 years	13	1	0	1	7.7
Breast cancer at any age	≥ 2 breast cancer <50 years	7	1	0	1	14.3
Breast cancer at any age	≥ 3 breast cancer at any age	9	0	3	3	33.3
Breast cancer at any age	≥ 1 ovarian cancer at any age	10	4	1	5	50.0
Breast and ovarian cancer	Any	5	2	1	3	60.0
Bilateral breast cancer	Any	25	4	1	5	20.0

### Risk prediction models (BOADICEA) and scoring methods (Manchester Scoring System)

**Figure 1:**
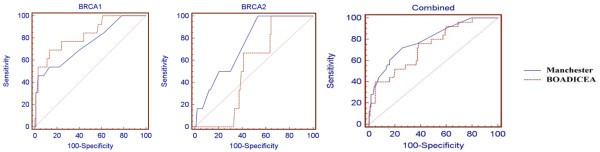
Receiver Operating Characteristic curves for Manchester Scoring and BOADICEA predictions of the probability of carrying a *BRCA1 *or *BRCA2 *mutation.

Using receiver operating curves (Figure 1), we evaluated the accuracy of using the Manchester Scoring System empirical method compared with the BOADICEA risk-prediction models to discriminate between those families that have a *BRCA *mutation and those that do not (Table 8). The classifications based on Manchester Score System and BOADICEA are similar for *BRCA1*, but significantly different for *BRCA2*, which BOADICEA did not predict accurately. In terms of discriminating between those with and without a mutation, the areas under the receiver operating curves, a common measure of the adequacy of a quantitative predictive algorithm, are 0.74 (95% confidence interval [CI] = 0.67 to 0.81) and 0.82 (95% CI = 0.75 to 0.88) for *BRCA1*, and 0.82 (95% CI = 0.75 to 0.88) and 0.56 (95% CI = 0.48 to 0.64) for *BRCA2*, for Manchester Score System and BOADICEA, respectively (Table 8).

**Table 8:** The sensitivities, specificities, and positive and negative predictive values calculated with a Manchester Scoring System cut-off score of ≥ 10 for each gene and ≥ 15 combined, and for BOADICEA score of ≥ 0.10 for each gene and ≥ 0.10 combined (ie, 10% probability of carrying a deleterious mutation)

	**Manchester Scoring System**			**BOADICEA**		
	
	***BRCA1***	***BRCA2***	**Combined**	***BRCA1***	***BRCA2***	**Combined**
**Area under the receiver operating curves**	0.74	0.82	0.80	0.82	0.56	0.73
**Standard error**	0.078	0.079	0.06	0.070	0.093	0.061
**95% CI**	0.67 – 0.81	0.75 – 0.88	0.72 – 0.86	0.75 – 0.88	0.48 – 0.64	0.65 – 0.80
**P value**^a^	0.0018	0.0001	0.0001	0.0001	0.507	0.0001
**Sensitivity**	57%	55%	72%	57%	9%	40%
**Specificity**	76%	87%	74%	93%	93%	85%
**Positive-predictive value**	20%	25%	37%	47%	10%	36%
**Negative-predictive value**	94%	96%	93%	95%	93%	87%

^a^P value is the probability that the sample area under the ROC curve is found when in fact, the true area under the ROC curve is 0.5 (i.e. null hypothesis: Area = 0.5).

The probability of identifying a mutation was 66% (96 of 145), 10% (15 of 145), 9.7% (14 of 145), 6.2% (nine of 145) and 7.6% (11 of 145), at ranges of scores 4 to 14, 15 to 16, 17 to 20, 21 to 24 and 25 and above by the Manchester Scoring System. Our analysis also indicated that a cut-off at a combined Manchester score of 15 seems optimal as a threshold (sensitivity of 72%, specificity of 74%, positive-predictive value of 37% and negative-predictive value of 93%). At a combined score of 18, the sensitivity was 56%, specificity was 84%, positive-predictive value was 42% and negative-predictive value was 90%.

For a combined score of 0.10 or above using BOADICEA, we observed sensitivity of 40%, specificity of 85%, positive-predictive value of 36% and negative-predictive value of 87%.

The total number of cases predicted by the Manchester Scoring System was eight *BRCA1 *mutation carriers, nine *BRCA2 *mutation carriers and 17 mutation carriers in total (using the probabilities listed [20,21]), and by BOADICEA was nine *BRCA1 *mutation carriers, five *BRCA2 *mutation carriers and 14 mutation carriers in total. This was compared with the observed numbers of 14 *BRCA1*, 11 *BRCA2 *and 25 total carriers (three *BRCA2 *carriers were among the 42 individuals excluded in the analysis because they had a single case of breast cancer and did not have any family history of cancer in the family). This suggests that both the Manchester Scoring System and BOADICEA methods underpredicted the probability of having a mutation in *BRCA1 *or *BRCA2 *in our cohort.

## Discussion

This study provides important data on the prevalence and spectrum of mutations in *BRCA1 *and *BRCA2 *in the multi-ethnic population of Malaysia. This key information was then coupled with the family history of cancer and selection criteria to determine the optimal strategy for clinical genetic testing in this population, keeping in mind that this service is not yet available in the national health care system and that it is necessary to target interventions at high-risk individuals who have the most health benefits to gain from available preventive and risk-reduction strategies.

### *BRCA1 *and *BRCA2 *mutation spectrum and prevalence

Of the 27 deleterious mutations identified, 13 mutations have never been previously reported in any other population. Two groups studying *BRCA *mutations in Singapore, where the population is also multi-ethnic but in different proportions (Chinese 77%, Malays 14% and Indians 8%), reported seven mutations in *BRCA1 *that were not observed in our cohort [24-27]. One mutation, c.2845insA in *BRCA1*, was recently reported to have a founder effect among the Malay population in Singapore [25,28,29], but this was not found in 44 Malays in our study.

We did not identify any mutations that occur with high frequency in Asians. Four mutations that have previously been reported in Asians: *BRCA1 *589delCT in at least four families from Southern China [30], *BRCA1 *1100delAT in families from Shanghai [16], *BRCA2 *4265delCT in at least three families from the Philippines [31] and *BRCA2 *2699del6 in two families from Indonesia [32], were also found in our cohort but these are unlikely to be common enough to warrant specific testing. Given the genetic diversity of the Asian populations, it is unlikely that screening for a panel of founder mutations will be as effective in this population as is the case for the Ashkenazi Jewish [33] or Icelandic [34] populations.

We found that *BRCA2 *may play an important role in genetic susceptibility among the small numbers of Malays in this cohort. Similar associations have been reported among the Filipinos and Japanese [31,35]. However, the reason for a higher proportion of *BRCA2 *mutations over *BRCA1 *mutations is not known, but may reflect cohort selection, genetic drift, or a possible prevalence of a modifying genetic or environmental factor that modifies the penetrance of *BRCA1 *or *BRCA2 *among Malays. Further studies using larger cohorts of Malay individuals are needed to address these possibilities.

In addition to the clearly deleterious protein-truncating mutations, a number of unclassified sequence variants were detected by sequencing. Of these, nine of 16 in *BRCA1 *(56%) and 17 of 31 in *BRCA2 *are novel (55%), and several of the remainder have only been described in Asian women. It is likely that the majority of these sequence variants have no clinical relevance, and the few that are likely to be deleterious are unlikely to change the basic conclusions of this study. We are conducting further analyses using established methods [36-38] to better understand the clinical significance of these sequence variants in our cohort.

### Predicting *BRCA1*- and *BRCA2*-positive family status in Asia

As *BRCA1 *and *BRCA2 *mutation testing is expensive, any ability to determine the probability that a specific family may benefit is an important issue. Several tools have been developed to help clinicians in predicting the probability of carrying a *BRCA1 *or *BRCA2 *mutation based on the familial history of breast and/or ovarian cancer and all tools have been based on populations where the age-standardised rate for breast cancer has been significantly higher than that reported in Malaysia (80 to 100 per 100,000 compared with 30 to 50 per 100,000 in Malaysia).

The Manchester Scoring System is perhaps the easiest of these prediction tools to use, and to determine whether the likelihood of identifying a mutation in a family reaches the 10% threshold for either *BRCA1 *or *BRCA2*. This model is does not require computer implementation. However, given that the Manchester Scoring System was devised using data from the Manchester region in northwest England, it is unclear whether it would be as effective in populations with different demographics, such as that in many Asian countries. Our analysis indicated that a cut-off at a combined Manchester score of 15 seems optimal as a threshold (sensitivity of 72%, specificity of 74%, negative-predictive value of 93%) but with a low positive-predictive value (37%). Notably, for the same cut-off of 15 among the Danish [39], the sensitivity was 84%, but the specificity was lower at 44%. The optimal cut-off appears to be lower for our population compared with studies in Australians [40] and French-Canadians in Canada [41], where comparable sensitivity and specificity (86% and 82% respectively in the Australians, and 72% and 64% respectively in the Canadians) were obtained with a higher score of 18. Taken together, our data suggests that in populations such as ours, where the overall incidence of breast and ovarian cancers is lower than that in Caucasian populations, family history is more significant therefore giving a higher specificity at a lower score.

In contrast, BOADICEA requires computation and is more difficult to implement in a clinical setting that is not familiar with risk prediction and genetic testing. Nevertheless, the strength of the model relies in its ability to compute extensive family history, and mutations in other genes relevant to breast cancer, such as *CHEK2*, to calculate the probability of carrying a mutation in the *BRCA *genes. In our analysis, a cut-off probability of 10% or higher yielded comparable specificity albeit with compromised sensitivity (85% and 40% respectively) to that reported in an analysis of 300 non-Ashkenazi Jewish, predominantly Caucasian families [42] (specificity 68% and sensitivity 73% respectively).

We found that both the Manchester Scoring System and BOADICEA underestimated the number of mutation carriers in our cohort. The most likely explanation, which is consistent with other studies, may be that both methods underestimate the number of carriers in families with insignificant family history [42], that is both methods underestimate the exceptional nature of the families, within the context of a lower population rate of breast cancer. In addition, the underestimate may be more significant for *BRCA2 *in BOADICEA because the allelic frequency of *BRCA2 *may be different in the ethnic population we have studied compared with Caucasian populations, and may be because *BRCA2 *may have a different penetrance in this population.

Overall, in this typically Asian cohort where 80% (149 of 187) of the breast cancer patients had no or only a single first- or second-degree relative with breast cancer or ovarian cancer, 15% of women carried deleterious mutations in *BRCA1 *or *BRCA2*. This suggests that a family history as reported by an individual who carries a mutation in *BRCA1 *or *BRCA2 *may be neither dramatic nor obvious. Indeed, in a study of 10,000 individuals in the USA, up to 13% of individuals with a single first- or second-degree relative with early-onset breast cancer or ovarian cancer, carried deleterious mutations [6]. This suggests that careful evaluation of paternal as well as maternal family history is required, especially in women diagnosed with breast cancer before age 50 years or ovarian cancer at any age, to enable the appropriate identification and counselling of individuals at risk of carrying mutations in *BRCA1 *and *BRCA2*. We believe that identification of individuals at risk is particularly significant in Asia because the problem of limited family structure is not uncommon, in part because of loss of family information from migration, and there remains significant stigma in talking about cancer [9].

## Conclusion

Taking into consideration the recommendations described above and our findings, it seems reasonable in a clinical setting to offer individuals with a personal or family history of cancer testing if they have a combined Manchester score of 15 or above. Lower thresholds could be used when resources become available and where the judgement of health care professionals involved in familial cancer clinics indicates. Our study underscores the need for larger collaborative studies among non-Caucasian populations to validate the role of genetic testing, the use of risk-prediction models and the role of risk-reducing strategies in ensuring that the other populations in the world may also benefit from the genomics and genetics era.

## Abbreviations

BIC = Breast Cancer Information Core; BOADICEA = Breast and Ovarian Analysis of Disease Incidence and Carrier Estimation Algorithm; CI = confidence interval; MLPA = multiple ligation dependent probe amplification

## Competing interests

The authors declare that they have no competing interests.

## Authors' contributions

ET, LSY, PK, DL, TGT and SS carried out the genetic studies, data collection and data analysis. ET, YSY, TMK, NAMT, YCH and TSH participated in the selection and recruitment of patients to the study. YCH and TSH conceived the study and ET and TSH drafted the manuscript. All authors read and approved the final manuscript.
